# The Effects of Hyperhydrating Supplements Containing Creatine and Glucose on Plasma Lipids and Insulin Sensitivity in Endurance-Trained Athletes

**DOI:** 10.1155/2015/352458

**Published:** 2015-06-17

**Authors:** Thelma P. Polyviou, Yannis P. Pitsiladis, Carlos Celis-Morales, Benjamin Brown, John R. Speakman, Dalia Malkova

**Affiliations:** ^1^Human Nutrition, School of Medicine, College of Medicine, Veterinary and Life Sciences, New Lister Building, Glasgow Royal Infirmary, 10-16 Alexandra Parade, Glasgow G31 2ER, UK; ^2^FIMS Reference Collaborating Centre of Sports Medicine for Anti-Doping Research, University of Brighton, Welkin House, 30 Carlisle Road, Eastbourne BN20 7SN, UK; ^3^BHF Glasgow Cardiovascular Research Centre, Institute of Cardiovascular & Medical Sciences, University of Glasgow, Glasgow G12 8TA, UK; ^4^Institute of Biological and Environmental Sciences, University of Aberdeen, Zoology Building, Tillydrone Avenue, Aberdeen AB24 2TZ, UK

## Abstract

The addition of carbohydrate (CHO) in the form of simple sugars to creatine (Cr) supplements is central. The study aimed to determine whether ingestion of glucose (Glu) simultaneously with Cr and glycerol (Cr/Gly) supplement is detrimental to plasma lipids of endurance-trained individuals and find out whether modification arising can be attenuated by replacing part of the Glu with alpha lipoic acid (Ala). Twenty-two endurance-trained cyclists were randomized to receive Cr/Gly/Glu (11.4 g Cr-H_2_O, 1 g Gly/kg BM, and 150 g Glu) or Cr/Gly/Glu/Ala (11.4 g Cr-H_2_O, 1 g Gly/kg BM, 100 g Glu, and 1 g Ala) for 7 days. Fasting concentration of TAG increased significantly (*P* < 0.01) after supplementation with Cr/Gly/Glu (before: 0.9 ± 0.2 mmol/L; after: 1.3 ± 0.4 mmol/L) and Cr/Gly/Glu/Ala (before: 0.8 ± 0.2 mmol/L; after: 1.2 ± 0.5 mmol/L) but changes were not different between the groups. Supplementation significantly (*P* < 0.05) increased the TAG to HDL-cholesterol ratio but had no effect on fasting concentration of total, HDL-, and LDL-cholesterol and insulin resistance. Thus, addition of Glu to Cr containing supplements enhances plasma TAG concentration and the TAG to HDL-cholesterol ratio and this enhancement cannot be attenuated by partial replacement of Glu with Ala.

## 1. Introduction

Creatine (Cr) is a guanidine compound which is naturally synthesised in the liver, kidney, and pancreas from amino acids arginine, glycine, and methionine [1]. Cr can also be obtained through diet, especially from meat and fish [2]. Dietary Cr is taken up to tissues, including skeletal muscle [3]. Enhancement in muscle Cr content facilitates growth in lean body mass, strength, and high intensity exercise performance [4]. It also enhances fluid retention and provides the improvement in thermoregulation during exercise in the heat [5, 6]. As a consequence of these findings, Cr supplementation has become popular amongst recreational and professional athletes.

*In vitro* [7] and* in vivo* [8] studies demonstrated that Cr uptake by rat skeletal muscle is increased by the presence of insulin. In humans, carbohydrate (CHO) ingestion, aimed at rising plasma insulin concentration, has been demonstrated to enhance both whole body Cr retention [9] and skeletal muscle Cr accumulation [10]. It has been suggested that muscle Cr accumulation is maximised when ~100 g of simple sugars is ingested with a typical 5 g dose of Cr supplement [11]. The amount of Glu required can be expected to increase available CHO intake above the habitually consumed level. Safety of such a high CHO intake while applying Cr supplementation protocols has not been investigated yet.

Some studies have demonstrated that increase in CHO intake may have detrimental impact on concentration of plasma lipids not only in sedentary [12–15] but also in well-trained individuals [16–18]. For example, in distance runners consumption of a high CHO diet for two weeks increased concentration of plasma triglycerides (TAG) and total and LDL-cholesterol and reduced concentration of HDL-cholesterol [16]. These results were supported by another study which examined the effects of 12-week high CHO diet on plasma lipids in 32 endurance-trained cyclists and found a significant increase in plasma concentration of total cholesterol and TAG [17]. A recent study also showed that in 9 trained males after 5 days on diet providing 70% of energy from CHO, plasma concentration of TAG was increased and concentration of HDL-cholesterol decreased [18]. Based on population studies, these changes would be expected to increase the risk of coronary heart disease [19].

Due to these possible detrimental changes in plasma lipids induced by high glucose (Glu) intake consumed with Cr containing supplements, there has been a search for alternative agents that stimulate insulin secretion and thus may be expected to enhance skeletal muscle Cr uptake. We previously demonstrated that the same increase in total body water and improvement in thermoregulatory and cardiovascular responses during exercise in the heat can be achieved when part of the Glu in the Cr/glycerol (Gly) hyperhydrating supplement is replaced with alpha lipoic acid (Ala) [20], a compound that has insulin-potentiating activity [21]. It remains unclear whether expected changes in plasma lipids can be attenuated by partial replacement of Glu with Ala.

This study aimed to determine whether ingestion of Glu simultaneously with Cr/Gly supplement is detrimental to the fasting concentration of plasma TAG and the TAG to HDL-cholesterol ratio, an independent risk factor of cardiometabolic risk [19, 22] of endurance-trained individuals, and find out whether modifications arising from Cr/Gly/Glu supplementation can be attenuated by replacing part of the Glu with Ala (Ala).

## 2. Methods

### 2.1. Participants

Twenty-two healthy endurance-trained males took part in the study. Physical characteristics of the participants are presented in Table 1. Eligibility was assessed via an interview and a medical questionnaire. During the interview, the investigator confirmed that participants had not been supplemented with Cr 6–8 weeks preceding the study, as this was an exclusion criterion. Nevertheless, the fact that previous Cr supplementation would act as an exclusion criterion was withheld from the participants. Upon recruitment, two participants were excluded from the Cr/Gly/Glu group as fasting plasma triglyceride concentration exceeded recommended healthy concentration of <1.7 mmol/L [23].

### 2.2. Study Design

Study participants were randomly assigned to receive Cr/Gly/Glu or Cr/Gly/Glu/Ala supplements for 7 days. Fasting blood samples were obtained and body weight was measured before and after 7 days of the supplementation. On the days of the blood sampling participants were also asked to provide a baseline urine sample and after this orally ingest a dose of deuterium oxide (D_2_O) (Ontario Hydro, Canada). Six hours after the D_2_O ingestion participants were asked to provide another urinary sample. During the 7 days preceding the supplementation and the 7 days of supplementation participants were asked to record all food and drink consumed.

### 2.3. Experimental Procedures

#### 2.3.1. Anthropometry and Body Composition

Measurements of body mass were taken using bioelectrical impedance scales (TBF-300, TANITA, Cranlea, UK). Height was determined using standard protocols [24].

#### 2.3.2. Supplementation

Subjects were matched for body mass and randomized in a double-blind fashion to receive Cr, Gly, and Glu or Cr, Gly, Glu, and Ala. Subjects were separated into two groups because of the long washout period associated with Cr [25]. The Cr/Gly/Glu group were instructed to ingest 20 g/day (4 × 5 g/day) of Cr monohydrate (Creapure Cr Monohydrate, Reflex Nutrition Ltd., UK), 2 g/kg of body mass per day (4 × 0.5 g/kg of body mass per day) of Gly (Glycerin, Care Plus, Huddersfield, UK), and 150 g/day (4 × 37.5 g/day) of Glu (SISGO Electrolyte Drink Powder, Ashwood Laboratories, Lancashire, England) and the Cr/Gly/Glu/Ala group were instructed to ingest 20 g/day (4 × 5 g/day) of Cr monohydrate (Creapure Cr Monohydrate, Reflex Nutrition Ltd., UK), 2 g/kg of body mass per day (4 × 0.5 g/kg of body mass per day) of Gly (Glycerin, Care Plus, Huddersfield, UK), 100 g/day (4 × 25 g/day) of Glu (SISGO Electrolyte Drink Powder, Ashwood Laboratories, Lancashire, England), and 1 g/d (4 × 250 mg/day) of Ala (Racemic mixture [R and S] Pure Bulk, USA). Daily intake of Cr, Gly, and Glu and Ala in Cr/Gly/Glu and Cr/Gly/Glu/Ala groups is presented in Table 2. Subjects were asked to avoid caffeine and alcohol intake for the full length of their participation in the study.

#### 2.3.3. Dietary Intake

For dietary intake records the participants were provided with food diaries and electronic scales (Salter Housewares, Kent, UK). Instructions of how to use the scales and how to complete properly the diaries were given to each participant individually. Participants were asked to record the amount of all the food and drinks consumed and report the time of consumption. They were also asked to record the amount of all food and drink which was left after eating. The dietary records were analysed for macronutrient and energy intake using dietary analysis software Windiets 2005 (The Robert Gordon University, Aberdeen, Scotland, UK). Amount of CHO provided by the supplements was added to amount of CHO provided by the diet. Since Gly is defined by the US Food and Drug Administration (FDA) as a CHO providing 4.32 kcal per gram, this was taken into consideration when calculating the amount of CHO consumed in the supplementation study.

#### 2.3.4. Blood Collection, Plasma Preparation, and Analysis

Subjects reported to the laboratory after an overnight 8-hour fast. On arrival to the laboratory subjects were asked to lay comfortably in a supine position for the duration of 10 minutes and then venous blood sample was collected in K_3_EDTA tube (BD, Vacutainer System, Franklin Lakes, NJ, USA). Tubes containing blood sample were immediately placed on ice and then centrifuged for 15 minutes at 3000 rpm (Hettich D-78532 Universal 320 R Centrifuge, Tuttlingen, Germany). Plasma was dispensed in 0.5 mL aliquots into labeled sterilised microcentrifuge cap tubes (red microcentrifuge tubes with cap, FR74073, Fischer Scientific, UK) and kept at −80°C until analyses.

Plasma concentration of TAG and total and HDL-cholesterol was analyzed by enzymatic colorimetric methods using commercially available kits (Roche Diagnostics GmbH, Mannheim, Germany). Plasma concentration of LDL-cholesterol was calculated using the Friedewald equation [26]. Glu was assayed using the hexokinase method, utilising a Roche kit (Roche Diagnostics GmbH, Mannheim, Germany). Glu, TAG, and total and HDL-cholesterol assays were performed on a Cobas Mira Plus (ABX Diagnostics, France). All samples for each participant were analysed in duplicate. The accuracy and precision of the assays was monitored using quality control sera (Roche Diagnostics GmbH, Mannheim, Germany; Randox Laboratories Ltd., Co., Antrim, Ireland; Kamiya Biomedical, Seattle, USA). The coefficients of variation were <3.1% for all calorimetric assays.

Plasma concentration of insulin was measured using an enzyme-linked immunoassay (ELISA) procedure based on a “sandwich” technique with the use of a commercially available ELISA kit following manufacturer instruction (Insulin ELISA kit 10-1113-01, Mercodia AB, Sylveniusgatan 8A, SE-754 50, Uppsala, Sweden). All samples for each participant were analysed in duplicate. The accuracy and precision of the assays was monitored using quality control sera (Diagnostic System Labs, TX, USA, and Linco Research Inc., St. Louis, MO, USA). Coefficients of variation were <4.0%. Fasting insulin and Glu were used for the assessment of insulin resistance by Homeostasis Model Assessment of Insulin Resistance (HOMA_IR_). For the insulin assay the coefficient of variation was <4.0%.

#### 2.3.5. Total Body Water (TBW) Determination

On arrival to the laboratory, participants provided a baseline urine sample and were then asked to orally ingest 0.5 g·kg^−1^ BM of D_2_O (Ontario Hydro, Canada). To evaluate the volume of isotopic distribution in body water, urine sample was collected again after 6 h. Both urine samples were collected in a dry plastic container. For purposes of analysis, the investigator transferred 2 mL from all urine samples from the dry plastic containers to glass vessels and stored them in −20°C. Urine samples were then analyzed by an isoprime isotope ratio mass spectrometer (Elementar Ltd., Manchester, UK), coupled to a Eurovector gas chromatograph (GC) fitted with an HT300A autosampler, as described elsewhere [27].

#### 2.3.6. Statistical Analysis

Data were assessed for normality of distribution using Anderson-Darling test. Statistical analysis was carried out using the 2-way ANOVA (i.e., before versus after supplementation for each treatment). Independent sample *t*-tests were used to examine whether changes induced in plasma lipids, Glu, and insulin resistance were different between the two treatments. Data was described as mean ± SD. All statistical analysis was carried out using SPSS for Windows version 17.0. Statistical significance was set at *P* ≤ 0.05.

#### 2.3.7. Ethical Considerations

All participants gave their informed consent and the study was approved by the Ethics Committee for Nonclinical Research Involving Human Subjects, University of Glasgow, and was performed according to the code of ethics of the World Medical Association (Declaration of Helsinki).

## 3. Results

Data on nutrient and energy intake during the 7 days preceding supplementation and the 7 days of supplementation are presented in Table 3. During the week preceding supplementation, there were no significant differences between Cr/Gly/Glu and Cr/Gly/Glu/Ala groups in energy and macronutrient intake. Being on both supplementation regimes significantly increased intake of available CHO (*P* < 0.001) and the percentage of energy provided by CHO (*P* < 0.001) while significantly reducing intake of fat (*P* < 0.01) and the percentage of energy obtained from fat (*P* < 0.01). During supplementation week, intake of starch (*P* < 0.01) was significantly lower whereas intake of sugars (*P* < 0.01) was significantly higher than that during the days of the habitual intake in both groups. Averaged daily energy intake, calculated by adding amount of Glu and Gly provided by supplements, was significantly (*P* < 0.01) higher during supplementation than during the presupplementation week but supplementation had no effect on body mass in the Cr/Gly/Gly (before: 70.0 ± 5.8 kg; after: 71.2 ± 5.3 kg, *P* > 0.05) and the Cr/Gly/Glu/Ala (before: 78.0 ± 8.5 kg; after: 79.2 ± 8.4 kg, *P* > 0.05) groups. On the other hand both types of supplementation induced significant (*P* < 0.05) increase in TBW (Cr/Gly/Gly: 1.7 ± 1.1 L; Cr/Gly/Glu/Ala: 1.2 ± 0.5 L, *P* < 0.05) and the increase in TBW tended (*r* = 0.40, *P* = 0.06) to correlate with the changes in body mass.

**Figure 1:**
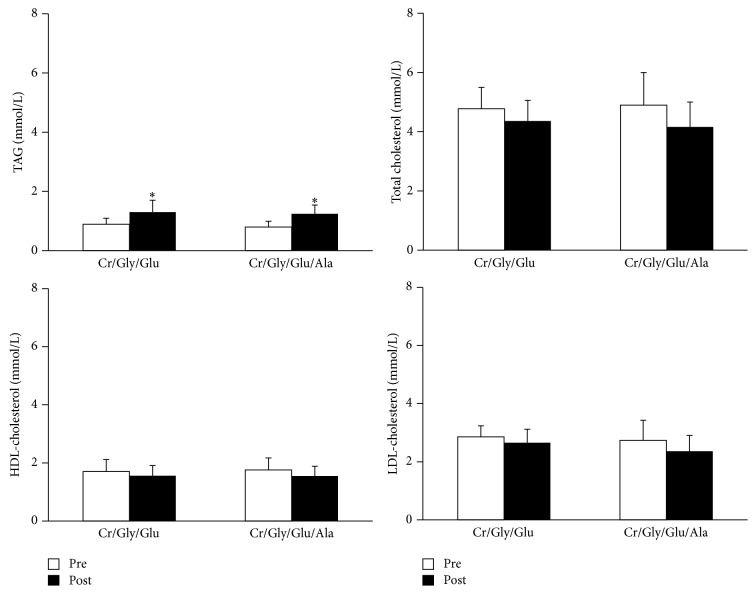
Fasting concentrations of plasma lipids at baseline (Pre) are represented with white bars and after 7 days of supplementation (Post) represented with black filled bars ^*∗*^Significantly different (*P* < 0.05) from presupplementation in corresponding group. Values are presented as means ± SD.

Fasting concentration of plasma lipids is presented in Figure 1. The concentration of TAG and total, LDL-, and HDL-cholesterol measured before supplementation was not significantly different between groups. Fasting concentration of plasma TAG increased significantly (*P* < 0.01) after supplementation with Cr/Gly/Glu (before: 0.90 ± 0.19 mmol/L; after: 1.30 ± 0.44 mmol/L, *P* < 0.01) and Cr/Gly/Glu/Ala (before: 0.77 ± 0.25 mmol/L; after: 1.2 ± 0.54 mmol/L, *P* < 0.01). The TAG to HDL-cholesterol ratio increased significantly following supplementation with Cr/Gly/Glu (before: 0.5 ± 0.07; after: 0.8 ± 0.19; *P* = 0.01) and Cr/Gly/Glu/Ala (before: 0.5 ± 0.1; after: 0.8 ± 0.2; *P* = 0.002). The increase in plasma TAG concentration and in the TAG to HDL-cholesterol ratio was not significantly different between groups. Supplementation had no effect on fasting concentration of total, HDL-, and LDL-cholesterol.

Before supplementation, the concentration of fasting Glu and insulin and insulin resistance evaluated from HOMA_IR_ were not significantly different between Cr/Gly/Glu and Cr/Gly/Glu/Ala groups. None of the supplements had impact on plasma Glu or insulin concentrations and HOMA_IR_ (Table 4).

## 4. Discussion

This study found that 7-day supplementation of endurance-trained individuals with hyperhydrating Cr/Gly supplement containing either 150 or 100 g of Glu significantly increased daily intake of available CHO and induced increase in fasting concentration of plasma TAG and the TAG to HDL-cholesterol ratio, an independent risk factor of cardiometabolic risk [19, 22]. We also found that these changes cannot be attenuated by replacing 50 g of Glu with Ala, another insulinotropic compound, required to promote Cr uptake by the skeletal muscle. We note that in endurance-trained athletes modified TAG concentrations remained below the unhealthy range [23] and suggest that increased intake of CHO during Cr supplementation does not represent a deleterious effect on cardiometabolic risk. However, in less trained individuals or habitual gym users often Cr loading when combined with Glu or other forms of simple sugars can be expected to increase plasma TAG concentration and the TAG to HDL-cholesterol ratio to higher levels. The latter is solely an extrapolation from our findings and may be worth investigating through future studies.

Our finding is that seven-day consumption of Cr based supplements containing Glu increased concentration not only in sedentary [28–31] but also in endurance-trained athletes. Plasma concentration of TAG is in consistency with the previous evidence showing that, in trained individuals, short-term enhanced CHO intake leads to significant increase in fasting plasma TAG concentrations [16–18]. The TAG to HDL-cholesterol ratio, an independent risk factor of cardiometabolic risk [22], has also increased significantly following supplementation with Cr/Gly/Glu and with Cr/Gly/Glu/Ala. Thus, our findings contribute to the notion that, regardless of endurance training being associated with efficient metabolism of plasma TAG and prolonged survival of HDL lipoproteins [32], endurance athletes cannot withstand the impact of acutely enhanced CHO intake. Thus, modification of macronutrient composition of the diet due to increased CHO intake enhances plasma TAG.

Previous studies show that Ala, a naturally existing compound that acts as a coenzyme for pyruvate dehydrogenase and *α*-ketoglutarate dehydrogenase, plays a key role in bridging glycolysis and the citric acid cycle and regulates Glu and lipid metabolism and has insulin-potentiating activity [21]. Thus, this study also aimed to find out whether changes of plasma lipids induced by Cr supplementation protocols, considering CHO addition, can be prevented or at least attenuated when 50 grams of Glu is replaced with Ala. We found that the extent of increase in plasma concentration of TAG was not significantly different between the group consuming 150 and the group consuming 100 g Glu plus Ala. This is not surprising since increase in CHO intake was significant and associated with reduction in fat intake in both groups. Thus, in both groups, diets consumed during the seven days of the supplementation period were high CHO/low fat diets and differ significantly from habitual diets. Our data suggest that diminishing daily Glu intake by 50 g and replacing that amount of Glu with Ala cannot prevent increase in plasma concentration of TAG induced by incorporation of Glu.

Prior supplementation plasma concentration of TAG was low and within a range of 0.9–1.2 and 0.4–1.2 mmol/L in the Cr/Gly/Glu and Cr/Gly/Glu/Ala groups, respectively, which is in agreement with other studies investigating lipid profiles of trained individuals [33]. After 7 days of consumption of both supplements, plasma TAG concentration was still below 1.5 mmol/L, a concentration which is known to have a detrimental impact on distribution of LDL subtractions, leading to the increase in proportion of LDL_3_ particles [34], the most atherogenic lipoprotein particles. In addition, regardless of other studies reporting that, in athletic individuals, increase in plasma TAG leads to a synergistic decrease in HDL-cholesterol [16–18] the present study found that, after 7 days of enhanced CHO intake, plasma concentration of HDL-cholesterol was not diminished. Thus, endurance-trained individuals with baseline concentration of plasma TAG being within healthy range [23] should be safe to enhance intake of simple sugars required for promotion of muscle Cr uptake. In addition, since TAG-raising effect of high CHO is transient and disappears with time [35], it could be suggested that short-term high intake of CHO should not be of any concern for healthy and well-trained individuals. This, however, may not be the case for less trained individuals such as habitual gym users who often combine Cr loading with enhanced intake of simple sugars and for sedentary overweight individuals in whom higher-CHO diets, particularly those enriched in refined CHO, create a metabolic state that promotes development of atherosclerosis [29, 36].

From the athletes' point of view increase in plasma TAG concentration found after both types of supplementation may be considered as beneficial rather than detrimental change. Indeed, the increase in plasma TAG concentration may be expected to increase VLDL-derived fatty acid availability and rates of fat oxidation during exercise and thus have a potential to slow the rate of glycogen utilization and delay the onset of fatigue during sport competition and training [31]. This hypothesis requires evaluation since in sedentary individuals short-term adaptation to a high CHO diet induces metabolic alterations suggestive of repartitioning fatty acids away from oxidation toward esterification in both liver and muscle [31]. In addition, increase in plasma TAG concentration and the TAG to HDL-cholesterol ratio due to Cr/Gly/Glu and Cr/Gly/Ala supplementation can be outweighed by the enhancement in muscle glycogen accumulation [37] and expansion of water compartments within the human body and thus attenuation in the rise in heart rate and reduction in thermal and cardiovascular strain during exercise in the heat without negatively impacting exercise economy [5].

Based on the data obtained from dietary records, we found that during the days of supplementation energy intake was higher than habitual energy intake. Thus, it could be argued that supplementation induced increase in plasma TAG concentration was a consequence not only of enhanced CHO intake but also of change in energy balance. However, both interventions had no significant impact on body mass, which implies that energy intake, during interventions, was not different from habitual energy intake. This implies that food intake recording might have been more accurate during supplementation days. Indeed, during interventions participants were contacted by the researchers daily as a means of enhancing compliance to interventions and also to remind participants to record food and drink intake. It is also important to note that only long-term energy imbalance leading to weight gain was found to be associated with an increase in plasma TAG concentrations [38], while acute dietary energy surplus (~3 MJ) had no effect on basal VLDL-TAG metabolism and thus fasting TAG concentration [39]. These finding are in consistency with recent data [40] suggesting that disturbance in energy balance leading up to more than 1 kg of visceral fat increase would be required to achieve changes in plasma TAG concentration found in our study. It is obvious that this increase in visceral or even total body fat did not take place in the current study. Indeed, our data demonstrate that body weight changes if any were exclusively related to the changes in TBW. Thus, an increase in plasma TAG concentration seen in both groups was most likely related to significant and quite extensive changes in macronutrient intake rather than changes in energy balance.

We found that insulin sensitivity as determined by HOMA_IR_ was not affected by supplementation. The expected adverse effects of high CHO intake on insulin sensitivity [41] could have been offset by the consumption of Cr and Ala, which have previously been found to improve insulin sensitivity. Ala supplementation has been shown to enhance activation of adenosine monophosphate activated kinase (AMPK), which subsequently enhanced insulin stimulated Glu disposal and reduced fasting insulin [42]. Similarly, Cr supplementation has been shown to improve Glu tolerance [43] in healthy males undergoing aerobic training [43]. It should be noted that due to very high cardiorespiratory fitness, participants of this study had very low HOMA_IR_ [44] and thus may have preserved insulin sensitivity regardless of high CHO intake. In addition there is evidence to suggest that increasing CHO intake improves rather than impairs glucose tolerance [45]. Thus, the adverse effects of acute increase in CHO intake on plasma lipid concentration can be expected to be balanced either by no effect or potentially by a favourable influence on Glu tolerance. We appreciate that not using the gold standard technique, euglycemic hyperinsulinemic clamp, for the assessment of insulin sensitivity and considering only fasting concentrations of insulin and Glu might have limited revealing impact on insulin and Glu dynamic.

## 5. Conclusion

In conclusion, in endurance-trained individuals, short-term increase in available CHO intake, through the Cr/Gly/Glu hyperhydrating supplement which has been used prior to exercise in the heat, causes a significant increase in plasma TAG concentration and the TAG to HDL-cholesterol ratio. These detrimental changes cannot be attenuated by partial replacement of Glu with insulin-potentiating agent such as Ala. These findings should be of potential interest not only to scientists but also to athletes, coaches, and sports dietitians.

## Figures and Tables

**Table 1:** Physical characteristics of participants in Cr/Gly/Glu (*n* = 11) and Cr/Gly/Glu/Ala (*n* = 11) groups. Data presented as mean ± SD.

	Cr/Gly/Glu	Cr/Gly/Glu/Ala
Age (y)	31 ± 10.5	32 ± 8.7
Height (cm)	177 ± 7.5	181 ± 5.4
Weight (kg)	71 ± 6.3	78 ± 8.4^*∗*^
BMI (kg/m^2^)	23 ± 1.8	24 ± 2.1
VO_2_ max (mL/kg/min)	61 ± 3.9	59 ± 4.5

^*∗*^Significantly different (*P* < 0.05) from Cr/Gly/Glu group.

**Table 2:** Daily intake of creatine (Cr), glycerol (Gly), and glucose (Glu) and alpha lipoic acid (Ala) in Cr/Gly/Glu and Cr/Gly/Glu/Ala groups.

	CR	Gly	Glu	Ala
Cr/Gly/Glu	20 (g)	1 g/kg/BM	150 (g)	—
Cr/Gly/Glu/Ala	20 (g)	1 g/kg/BM	100 (g)	1000 (mg)

**Table 3:** Averaged daily energy, carbohydrate (CHO), fat, and protein intake in Cr/Gly/Glu and Cr/Gly/Glu/Ala groups before (habitual diet) and during 7 days of supplementation. Values are mean ± SD.

	Cr/Gly/Glu		Cr/Gly/Glu/Ala	
	Before	During	Before	During
Available CHO (g)	470 ± 115	613 ± 61^*∗*^	376 ± 88	514 ± 50^*∗*^
Sugar (g)	161 ± 52	389 ± 36^*∗*^	128 ± 43	325 ± 21^*∗*^
NMES (g)	86 ± 14	58 ± 15^a^	69 ± 39	35 ± 13^a^
Starch (g)	226 ± 67	178 ± 44^a^	248 ± 172	158 ± 30^a^
Fibre (g)	22 ± 8	17 ± 7	19 ± 4	16 ± 6
Fat (g)	104 ± 38	69 ± 11^a^	101 ± 28	83 ± 27^a^
Protein (g)	86 ± 16	101 ± 15	114 ± 29	112 ± 31
Available CHO (g/kg/BM)	7 ± 2	9 ± 2^*∗*^	5 ± 1	7 ± 1^*∗*^
Energy (MJ/day)	10 ± 2	14 ± 1^*∗*^	11 ± 1	14 ± 2^*∗*^
From CHO (%)	53 ± 4	68 ± 2^*∗*^	50 ± 10	60 ± 5^*∗*^
From fat (%)	37 ± 6	19 ± 2^a^	35 ± 7	24 ± 9^a^
From protein (%)	14 ± 3	20 ± 8	12 ± 2^a^	18 ± 4

*Note:* NMES: non-milk extrinsic sugars. ^*∗*^Significantly (*P* < 0.01) higher than before; ^a^significantly (*P* < 0.01) lower than before.

**Table 4:** Plasma concentration of fasting glucose and insulin, and homeostasis model assessment of insulin resistance (HOMA_IR_) preceding supplementation (Pre) and after 7 days of supplementation (Post). Values are means ± SD.

	Cr/Gly/Glu		Ala /Cr/Gly/Glu	
	Pre	Post	Pre	Post
Glucose (mmol/L)	4.8 ± 0.4	4.9 ± 0.2	4.9 ± 0.5	4.9 ± 0.4
Insulin (*μ*lU/mL)	2.8 ± 0.4	2.9 ± 0.6	2.6 ± 0.6	2.8 ± 0.6
HOMA_IR_	0.6 ± 0.1	0.6 ± 0.1	0.6 ± 0.2	0.6 ± 0.2
